# SLIViT: a general AI framework for clinical-feature diagnosis from limited 3D biomedical-imaging data

**DOI:** 10.21203/rs.3.rs-3044914/v2

**Published:** 2023-11-21

**Authors:** Oren Avram, Berkin Durmus, Nadav Rakocz, Giulia Corradetti, Ulzee An, Muneeswar G. Nitalla, Akos Rudas, Yu Wakatsuki, Kazutaka Hirabayashi, Swetha Velaga, Liran Tiosano, Federico Corvi, Aditya Verma, Ayesha Karamat, Sophiana Lindenberg, Deniz Oncel, Louay Almidani, Victoria Hull, Sohaib Fasih-Ahmad, Houri Esmaeilkhanian, Charles C. Wykoff, Elior Rahmani, Corey W. Arnold, Bolei Zhou, Noah Zaitlen, Ilan Gronau, Sriram Sankararaman, Jeffrey N. Chiang, Srinivas R. Sadda, Eran Halperin

**Affiliations:** 1 Department of Computational Medicine, University of California Los Angeles, Los Angeles, California, United States of America; 2 Department of Computer Science, University of California Los Angeles, Los Angeles, California, United States of America; 3 Department of Anesthesiology and Perioperative Medicine, University of California Los Angeles, Los Angeles, California, United States of America; 4 Doheny Eye Institute, Pasadena, California, United States of America; 5 Department of Ophthalmology, University of California Los Angeles, Los Angeles, California, United States of America; 6 Department of Ophthalmology and Visual Sciences, University of Louisville, Kentucky, United States of America; 7 Retina Consultants of Texas, Retina Consultants of America, Houston, Texas; 8 Departments of Radiology, University of California Los Angeles, Los Angeles, California, United States of America; 9 Departments of Bioengineering, University of California Los Angeles, Los Angeles, California, United States of America; 10 Departments of Pathology, University of California Los Angeles, Los Angeles, California, United States of America; 11 Department of Neurology, University of California Los Angeles, Los Angeles, California, United States of America; 12 Department of Human Genetics, University of California Los Angeles, Los Angeles, California, United States of America; 13 School of Computer Science, Reichman University, Herzliya, Israel

## Abstract

We present SLIViT, a deep-learning framework that accurately measures disease-related risk factors in volumetric biomedical imaging, such as magnetic resonance imaging (MRI) scans, optical coherence tomography (OCT) scans, and ultrasound videos. To evaluate SLIViT, we applied it to five different datasets of these three different data modalities tackling seven learning tasks (including both classification and regression) and found that it consistently and significantly outperforms domain-specific state-of-the-art models, typically improving performance (ROC AUC or correlation) by 0.1–0.4. Notably, compared to existing approaches, SLIViT can be applied even when only a small number of annotated training samples is available, which is often a constraint in medical applications. When trained on less than 700 annotated volumes, SLIViT obtained accuracy comparable to trained clinical specialists while reducing annotation time by a factor of 5,000 demonstrating its utility to automate and expedite ongoing research and other practical clinical scenarios.

Biomedical imaging analysis is a critical component of clinical care with widespread use across multiple domains. For example, analyzing optical coherence tomography (OCT) images of the retina allows ophthalmologists to diagnose and follow up on ocular diseases, such as age-related macular degeneration (AMD), and tailor appropriate and personalized interventions to delay the progression of retinal atrophy and irreversible vision loss^1,2^. Another example is the analysis of heart function using cardiac imaging, such as heart computed tomography (CT) and ultrasound. Monitoring heart function can help cardiologists assess potential cardiac issues, prescribe medications to improve a medical condition, e.g., reduced heart ejection fraction, and guide treatment decisions^3,4^. Lastly, radiologists’ analysis and regular monitoring of breast imaging such as mammography and magnetic resonance imaging (MRI) help detect early breast cancers, initiate a consequent interventive therapy, and determine the effectiveness of such therapeutics^5,6^. These medical insights and actionable information are obtained following an expert’s time-intensive manual analysis. The automation of these analyses using artificial intelligence may further improve healthcare as it reduces costs and treatment burden^7^.

Deep vision models, such as Convolutional Neural Networks (CNNs) and their derivatives, are considered state-of-the-art methods to tackle computer vision tasks in general^8,9^ and medical-related vision tasks in particular^10^. In order to train a deep vision model to accurately learn and predict a target variable in a general vision task (excluding segmentation tasks) from scratch, a very large number of training samples is needed. Transfer learning addresses this challenge by pre-training a vision model for a general learning task on a very large data set, and then using this general model as a starting point for training a specialized model on a much smaller dataset^11^. The key advantage of transfer learning is that the pre-training can be done on a large dataset in another domain, where data are abundant, and then the fine-tuning can be done using a small dataset in the domain of interest. Using a transfer learning approach, a plethora of previously developed deep vision methods analyzing 2D biomedical-imaging^12–15^, were first pre-trained on over a million labeled natural images (in a supervised fashion) taken from ImageNet^16^, and later on, fine-tuned to a specific medical-learning task on a much smaller number of labeled biomedical images (typically fewer than 10,000). Some methods used self-supervised-based transfer-learning techniques relying mainly on unlabeled medical data^17–19^, and others combined both natural and medical images^7,20^. Overall, the understanding that pre-trained weights can be leveraged as ‘prior knowledge’ for fine-tuning downstream learning tasks, were major factors in the fruitfulness of the majority of these 2D biomedical-imaging deep vision models.

Many diagnoses rely, however, on volumetric biomedical imaging (e.g., 3D OCT and MRI scans, or ultrasound videos) and transfer learning is not directly applicable, since in contrast to the 2D domain, there is no large annotated ‘ImageNet-like’ dataset of structured 3D scans. Moreover, annotating 3D biomedical images is far more labor-prohibitive than 2D images. For example, a 3D OCT scan that is composed of 97 2D frames (usually referred to as B-scans) usually requires a 5–10 minutes inspection of a highly trained clinical retina specialist in order to detect retinal-disease biomarkers, such as, the volume of a drusen lesion^21^. Therefore, considering the resources typically devoted to such a task, it is practically infeasible to annotate 100,000 (or more) volumes, to eliminate the necessity of supervised transfer learning. In fact, even merely compiling such large-sized volumetric datasets (without labels) that is required for self-supervised-based learning^22^ could be cost-, processing-, and storage-prohibitive when standard resources are available^23^. These gaps are acute because state-of-the-art supervised models for 3D image analysis, such as 3D ResNet^24^ and 3D Vision Transformer^25^ (ViT), involve the optimization of a very large number of parameters, thus requiring large datasets for training^26^.

Nonetheless, several attempts were undertaken to tackle volumetric-biomedical-imaging learning tasks with sparsely annotated training datasets on different data modalities. For instance, SLIVER-net was designed for binary classification of AMD biomarkers in 3D OCT scans^27^. EchoNet was designed to predict heart ejection fraction (EF) in echocardiograms^28^. A few other recent studies achieved state-of-the-art performance using 2D-Slice-CNN-based methods and 3D ResNet-based architectures in diagnosing Alzheimer’s disease^29^, breast cancer^30^, and Parkinson’s disease^31^ in 3D MRI scans. Notably, although 3D ResNet was first published in 2018, it is still largely considered a solid baseline and evidently, very popular not only on MRI studies (e.g., ^30,31^), but also across other recent volumetric-medical-imaging-modality studies such as ultrasound^32^ and CT^33^ studies. The main limitation of each of these approaches is that they are all tailored and optimized for specific biomedical data modality and domain. While each data modality requires a specific treatment, there are commonalities across the different data modalities, and a foundational approach that can provide improved results across multiple modalities will provide a faster development time for future predictive models. UniMiSS, a pioneering pyramid U-like Medical Transformer devised by Xie Y., et al.^19^, has recently been proposed to tackle this gap by utilizing multi-modal unlabeled medical images in a self-supervised manner. UniMiSS surpassed a diverse set of strong self-supervised approaches^34–38^ in a variety of medical-imaging learning tasks with different data modalities. However, with respect to volumetric imaging, it was tested on a single classification problem in a single imaging modality, and regression was not addressed at all. Thus, the full utility of transfer learning has yet to be attained across different modalities of volumetric-medical-imaging technologies.

Here, we present the SLice Integration by Vision Transformer (SLIViT) framework, a uniform 3D-based deep-learning model that overcomes the annotation bottleneck and is adept at volumetric-biomedical-imaging learning tasks. We leverage the combination of a 2D ConvNeXt-based^39^ feature-map extractor and a tweaked ViT^40^ together with cross-dimension and cross-domain (i.e., imaging modality, organ, and pathology) transfer learning. The 2D-based feature-map extractor allows leveraging prior 2D biomedical (and non-biomedical) vision knowledge when extracting information from a given volume in a variety of medical-imaging modalities. The attention-based mechanism of the ViT allows next to integrate the extracted information across the 2D frames of the volume in question.

Specifically, we demonstrate the generalizability and utility of SLIViT in very different medical domains, including retinal-disease risk biomarkers diagnosis in 3D OCT scans, cardiac function in echocardiogram videos, and hepatic-disease severity assessment from 3D MRI scans. We show that SLIViT consistently attains significantly improved performance compared to both strong generic baselines and domain-specific state-of-the-art models. Notably, the architecture and hyperparameters stay invariant across (tasks and) data modalities, that is, SLIViT provides these improved performance results across data modalities with neither tailoring the architecture nor optimizing hyperparameters per (task or) data modality, unlike other medical-imaging learning methods(e.g., ^7,13,19^). We further demonstrate that SLIViT’s performance is comparable to clinical specialists’ manual annotation, and that it shortens the annotation time by a factor of 5,000; hence it can potentially be used to save resources, reduce the burden on clinicians, and expedite ongoing research^7^. Finally, we demonstrate that SLIViT is robust to frame permutation, suggesting that (1) it is able to reconstruct long-range dependencies of the volume’s depth dimension (that are likely ignored when the volume is tiled; see next section); and (2) it could be applied to datasets in which the slice order (within a volume) is not recorded, a recurring situation in currently available public limited datasets. Of note, compared to other methods (e.g., ^19^), SLIViT does not require task-specific hyperparameter tuning and is relatively memory-thrifty (and thus can be effectively trained using standard hardware in reasonable time). Both ultimately facilitate generalizability, reproducibility, and successful applicability by a broader community of researchers to their datasets.

## Results

### A unified AI framework for analyzing volumetric biomedical-imaging data

**Figure 1 |:**
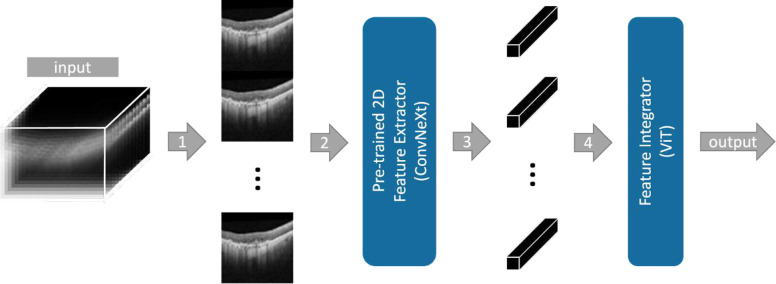
The proposed SLIViT framework The input of SLIViT is a 3D volume of N frames of size HxW. (1) The frames of the volume are resized and vertically tiled into an “elongated image”. (2) The elongated image is fed into a ConvNeXt-based Feature Extractor that was pre-trained on both natural and medical 2D labeled images. (3) N feature maps are extracted (each corresponding to an original frame). (4) Feature maps are (tokenized and) fed into a ViT-based Feature Integrator followed by a fully-connected layer that outputs the prediction for the task in question.

In this study, we devise SLIViT, a deep-learning vision model for automatic annotation of medical features in three-dimensional biomedical images. An overview of SLIViT is summarized in Figure 1. SLIViT preprocesses volumes into 2D images and then combines two deep vision architectures: (1) a ConvNeXt backbone module^39^ that extracts feature maps for the slices (i.e., 2D frames of a volume), and (2) a ViT module^40^ that integrates the slices feature maps into a single diagnosis prediction. One key part of SLIViT is that its feature-map extractor is initialized by pre-trained weights. These weights were obtained by pre-training a 2D ConvNeXt (T variant) first on ImageNet^16^ and then on an independent 2D OCT B-scan dataset, compiled by Kermany DS., et al.^41^, and labeled with retinal-disease coarse risk factors. These pre-trained weights, that were used for initialization on each of the experiments detailed in this study, allowed SLIViT to improve the performance in a variety of learning tasks especially when a very small training dataset is available (few hundreds of samples). Our hypothesis was that the basic features that are extracted from 2D B-scans when learning one task could serve as an improved training starting point not only for 3D OCT scans but also for other data types, such as ultrasound video or 3D MRI.

In order to cope with volumetric data, we treat each volume as a set of slices. A similar technique was shown to be effective for volumetric data modalities^42^. Essentially, each original slice of the volume is embedded into a single feature map. However, SLIViT reduces memory overhead and accelerates the processing time, by tiling the 2D images into a single elongated 2D image (rather than a set of separate images), such that it conforms with the input dimension expected by the 2D-based feature-map extractor. Once the feature maps are extracted, they are paired with (trainable) positional embeddings and comprehensively aggregated using a downstream ViT module^40^. SLIViT’s ViT module together with (trainable) positional embeddings allow to preserve the long-range dependencies across the depth dimension if needed^29,43^. Similar divide-and-conquer schemes were shown to be fruitful in other studies as well^44,45^. Of note, the ViT’s attention mechanism implicitly eliminates the necessity for image registration preprocessing.

**Figure 2 |:**
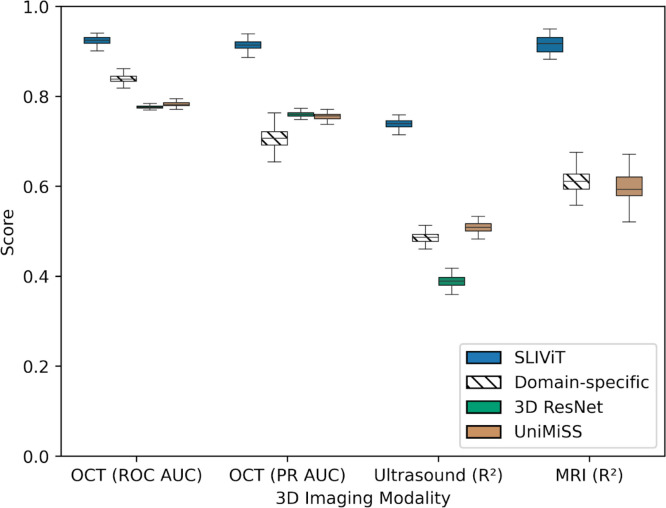
SLIViT’s outperformance overview Shown are the performance scores in one classification task (with two different metrics) of eye disease biomarker diagnosis in volumetric-OCT scans and two regression tasks of (1) heart function analysis in ultrasound videos and (2) liver fat levels imputation in volumetric MRI scans. Domain-specific methods (hatched) used are SLIVER-net, EchoNet, and 3D ResNet, for OCT, ultrasound, and MRI, respectively. The general cross-modality benchmarking used are 3D ResNet (green) and UniMiSS (brown) which are (fully) supervised and self-supervised-based, respectively (see relevant experiment’s section for additional benchmarking). Box plot whiskers represent a 90% CI.

We tested SLIViT on five datasets of three different data modalities (OCT, ultrasound, and MRI) with a limited number of annotated samples, tackling a variety of clinical-feature learning tasks (including both classification and regression). In the OCT experiment, we evaluated the diagnosis performance of ocular disease high-risk factors^27^ and measured it by both the receiver operating characteristic (ROC) area under the curve (AUC) and precision-recall (PR) AUC. In the ultrasound and MRI experiments, we compared the *R*^2^ of the models’ predictions vs. ground truth in (respectively) cardiac function analysis and in hepatic fat level imputation. In each data modality, we compared SLIViT with a diverse set of up to six strong baselines, including domain-specific^24,27–29^ and generic (fully-supervised-^24,25^ and self-supervised-based^7,19^) state-of-the-art methods. SLIViT manifested consistent and significant performance superiority across domains (Fig. 2). In the following sections we present these and additional results in detail.

### SLIViT outperforms state-of-the-art models in detecting ocular disease high-risk factors using 3D OCT scans

**Figure 3 |:**
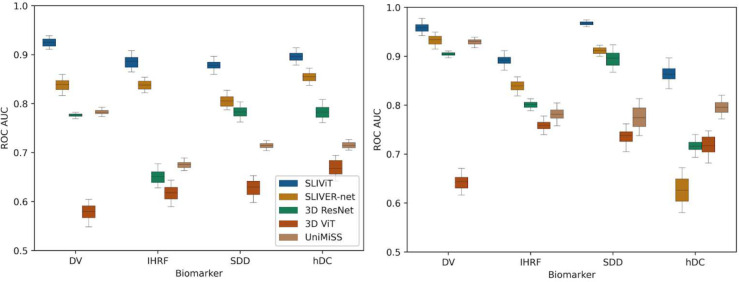
ROC AUC performance comparison of five models in four independent AMD-biomarker classification tasks when trained on less than 700 OCT volumes Shown are the ROC AUC scores of SLIViT (blue), SLIVER-net (orange), 3D ResNet (green), 3D ViT (red), and UniMiSS (brown) on four single-task classification problems of AMD high-risk factors in two independent volumetric-OCT datasets. The expected performance of a naive classifier is 0.5. The left panel shows the performance when trained and tested on the Houston Dataset. The right panel shows the performance when trained on the Houston Dataset and tested on the SLIVER-net Dataset (see Table S1A). Box plot whiskers represent a 90% CI.

We first compared SLIViT’s performance against trained SLIVER-net (subjected to the same pre-training approach), 3D ResNet, 3D ViT, and UniMiSS models, on the Houston Dataset which includes only 691 OCT B-scan volumes of different individuals (see Methods). OCT B-scan volume data were collected from independent individuals affected in at least one eye by dry AMD, a globally leading cause of irreversible central visual impairment^46^. Each OCT volume had four different binary labels of AMD high-risk biomarkers- drusen volume larger than 0.03 *mm*^*3*^ (DV), intraretinal hyperreflective foci (IHRF), subretinal drusen deposits (SDD), and hyporeflective drusen cores (hDC)^47^. The annotation was done by a senior retina specialist and the procured positive-label frequencies of DV, IHRF, SDD, and hDC, were 47%, 43.5%, 52.8%, and 31.3%, respectively. We randomly split the dataset into train, validation, and test sets of sizes 483 (70%), 104 (15%), and 104 (15%), respectively, and trained four different SLIViT models (one per binary label). We used both ROC AUC and PR AUC scores (the latter is also known as average precision or average positive predictive value) for performance evaluation. The models were trained (using less than 600 volumes) and tested on the same split (see left panels of Figures 3 and S1, and Table S1). In all four biomarkers, SLIViT significantly outperformed the other approaches in both evaluation metrics. For example, in the DV classification task (also shown as the OCT experiment in Fig. 2) SLIViT (ROC AUC=0.924; CI [0.909, 0.938]) was significantly better compared to the second-best performing method (SLIVER-net ROC AUC=0.838; CI [0.813, 0.86]; paired t-test p-value<0.001). In terms of average precision of the DV classification, SLIViT (PR AUC=0.914; CI [0.898, 0.928]) significantly outperformed the second-best performing method (3D ResNet PR AUC=0.759; CI [0.748, 0.769]; paired t-test p-value<0.001). Notably, since the biomarkers considered in these experiments are all structural, their identification requires aggregation of three-dimensional information. Thus, the ability of SLIViT to successfully identify these biomarkers suggests that it adequately captures a three-dimensional signal within a given volume.

To further challenge SLIViT we sought to explore its performance on the SLIVER-net Dataset used in the original SLIVER-net study^27^. In this task, SLIVER-net should have an advantage as it was optimized for this dataset. The SLIVER-net Dataset was composed of roughly one thousand OCT scans (imaged from independent individuals in an Amish population) collected from three different clinical centers (see Methods). We trained SLIViT, SLIVER-net (subjected to the same pre-training approach), 3D ResNet, 3D ViT, and UniMiSS, this time using all the 691 Houston Dataset volumes and used the SLIVER-net Dataset as the test set. For some biomarker classification tasks, the relative improvement of SLIViT compared to SLIVER-net was reduced, as expected in this setting. Yet, SLIViT was never overperformed by the other approaches, in any of the four AMD-biomarker classification tasks (see right panels of Figures 3 and S1, and Table S1).

### SLIViT outperforms state-of-the-art models in analyzing cardiac function using ultrasound videos

**Figure 4 |:**
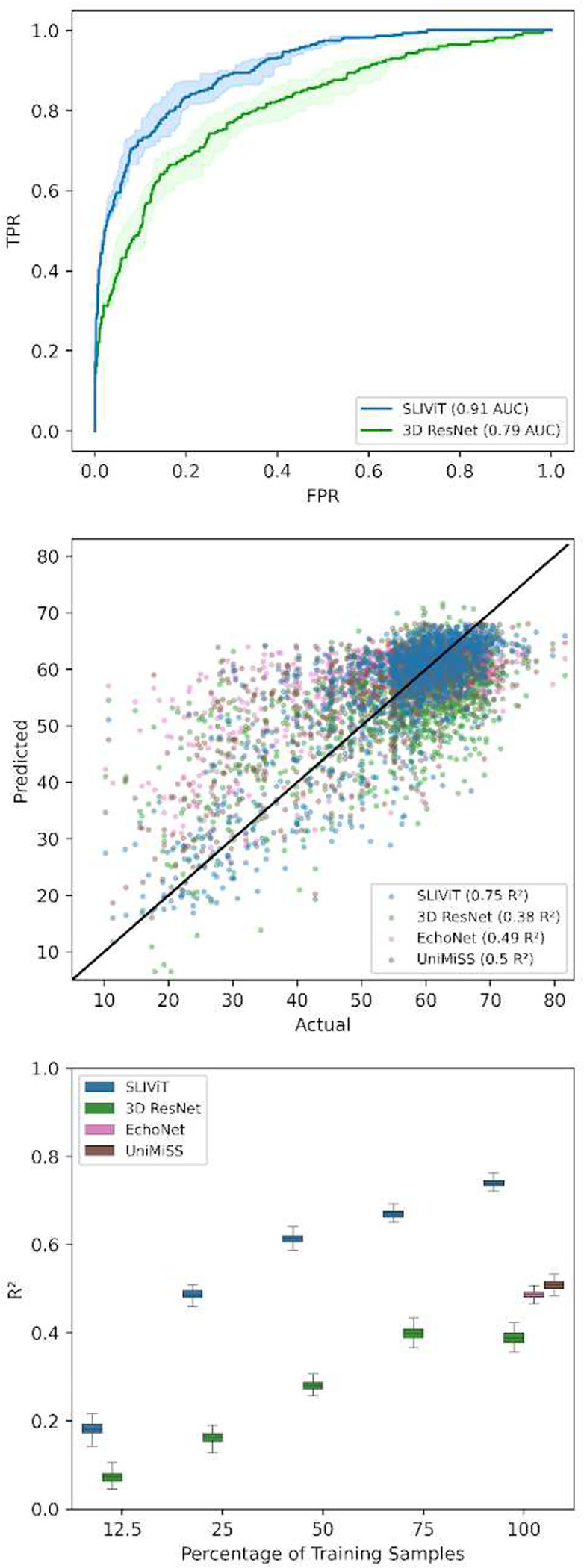
Performance comparison on cardiac function prediction tasks using echocardiograms Upper panel - ROC curves of cardiomyopathy prediction (EF<0.5). Middle panel - predicted vs. actual EF levels for three different models trained on the original training set (solid black line represents the y=x line). Lower panel- *R*^2^ performance of heart EF prediction using different percentages of the original training dataset. Box plot whiskers represent a 90% CI. Of note, when SLIViT was trained on 25% (n=1,866) of the original training set it obtained similar accuracy as the other examined methods when trained on 100% (n=7,465) of the training set.

In order to evaluate SLIViT’s generalizability, we next tested it on other 3D data modalities. The EchoNet-Dynamic Dataset contains 10,030 standard apical four-chamber view ultrasound videos (echocardiograms) obtained from unrelated individuals, each associated with a continuous number representing the corresponding ejection fraction (EF) measured in a clinical setting^48^. The EF is measured by tracing the chamber volume of the left ventricle in the end-systole and end-diastole, and is a key metric of cardiac function as it measures how well the heart’s left ventricle is pumping blood. Low EF measurements (<0.5) can indicate cardiomyopathy or other heart problems^3,49^. As a first experiment, we sought to explore SLIViT’s ability to predict cardiomyopathy as a binary classification task. To this end, we binarized the EF measurements accordingly (>=0.5 was considered as normal^50,51^) and, using the original EchoNet-Dynamic Dataset split, trained SLIViT and 3D ResNet (Fig. 4, upper panel). SLIViT obtained 0.913 ROC AUC (CI [0.901, 0.928]) and significantly overperformed 3D ResNet with 0.793 ROC AUC (CI [0.772, 0.814]) (paired t-test p-value<0.001).

In a second experiment, we sought to test SLIViT in a regression task. Previously, Ghorbani A., et al., implemented EchoNet, which is a GoogLeNet-based architecture for predicting the EF of a given echocardiogram video, and obtained a 0.5 *R*^2^ on the EchoNet-Dynamic Dataset test set^28^. This reported result did not include a CI (that would allow a direct comparison) and the trained model itself was not published. Thus, we implemented the proposed method and were able to reproduce similar levels of performance (*R*^2^=0.489; CI [0.434, 0.526]). Using the same split from the original EchoNet paper, we then trained SLIViT and obtained a significant improvement of 0.75 *R*^2^ (CI [0.706, 0.781]; paired t-test p-value<0.001). A scatter plot of the actual-versus-predicted per trained model is shown in the middle panel of Fig. 4. As we did in all other experiments, we also tested 3D ResNet and UniMiSS and observed that both significantly underperformed SLIViT with 0.384 (CI [0.364, 0.413]) and 0.502 (CI [0.487, 0.531]) *R*^2^, respectively (see ultrasound experiment in Fig. 2 and middle and lower panels of Fig. 4). Moreover, we also examined (1) a factorized spatiotemporal ResNet architecture (R(2+1)D, in contrast to the 3D-filter-based R3D ResNet we used across the study) that is known to capture well both spatial and temporal features from video frames and achieved state-of-the-art performance in a variety of video-based learning tasks^24^, and (2) 3D ViT^25^ Both methods performed below par compared to the other abovementioned benchmarks (*R*^2^=−0.081; CI [−0.106, −0.056] and *R*^2^=0.333; CI [0.27, 0.396], respectively).

This result, together with the exceptional magnitude of this public annotated dataset, further motivated us to examine the dynamics of the training set size and SLIViT’s performance in predicting the EF of a given echocardiogram (Fig. 4, lower panel). We randomly sampled size-decreasing subsets from the original training set and trained a SLIViT model per subset. Compared to other examined methods trained on the original training set (n=7,465), when SLIViT used the 25% subset (n=1,866) its performance (*R*^2^=0.487; CI [0.466, 0.507]) was significantly better than R3D, R(2+1)D, and 3D ViT (paired t-test p-value<0.001); on par with EchoNet (paired t-test p-value>0.579); and significantly lower than UniMiSS (paired t-test p-value<0.001). When SLIViT used the 50% subset, it significantly outperformed all other benchmarked methods (*R*^2^=0.614; CI [0.594, 0.634]; paired t-test p-value<0.001). These observations substantiate SLIViT’s ability to appropriately learn spatiotemporal features using a sparsely-labeled dataset.

### SLIViT outperforms state-of-the-art models in predicting hepatic fat levels in 3D MRI scans

We next sought to evaluate SLIViT ability to model 3D MRI data. We used a UK Biobank Dataset containing 3D hepatic MRI scans and a corresponding measurement for hepatic proton density fat fraction (PDFF) level. The PDFF measurement provides an accurate estimation of hepatic fat levels and it is also proposed to be used as a non-invasive method to limit unnecessary hepatic biopsies^52–54^. The development of a quantitative measurement of fat has been instrumental in improving the diagnosis of various fatty-liver and diabetes-related diseases^55–59^. We removed unlabeled scans and preprocessed the rest of the dataset to contain only a single scan per individual. In this experiment we compared SLIViT to 3D ResNet (that plays a double role- both the general and domain-specific state-of-the-art method^29–31^) and UniMiSS. We randomly split the dataset and trained both models to measure PDFF levels of a given 3D MRI. SLIViT reached 0.916 *R*^2^ (CI [0.879, 0.952]) and significantly outperformed both 3D ResNet and UniMiSS that obtained 0.611 (CI [0.566, 0.644]) and 0.599 (CI [0.531, 0.667]) *R*^2^, respectively (paired t-test p-value<0.001; See MRI experiment in Fig. 2). We also evaluated the performance of 3D ViT and a recently developed 2D-Slice-CNN-based architecture, that was shown to perform well on volumetric-MRI learning tasks^29^, but they both ended up with poor performance compared to all the abovementioned benchmarks (*R*^2^=0.18 (CI [0.145, 0.214]) and −0.130 (CI [−0.111, −0.148]), respectively).

### SLIViT efficiently attains the quality of clinical specialists

**Figure 5 |:**
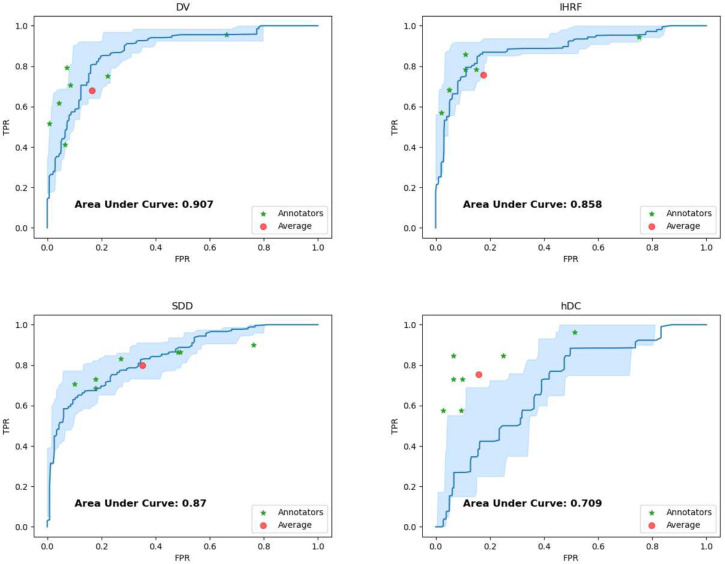
SLIViT’s ROC curve compared to junior clinical retina specialists’ assessment Shown are the ROC curves (blue) of SLIViT trained to predict four AMD high-risk biomarkers (DV, IHRF, SDD, and hDC; see main text) using less than 700 OCT volumes (Houston Dataset) and tested on an independent dataset (Pasadena Dataset). The light-blue shaded area represents a 90% CI for SLIViT’s performance. The red dot represents the specialists’ average performance. The green asterisks correspond to the retina specialists’ assessments. Two of the clinical specialists obtained the exact same performance score for IHRF classification.

To showcase the potential utility of automating the detection of AMD high-risk biomarkers we gathered the Pasadena Dataset, a third 3D OCT dataset containing 205 3D OCT volumes of (205) independent individuals. The ground truth for this dataset was obtained by three senior retina specialists (we used a majority vote when there was no consensus). We asked seven junior clinicians to (independently) annotate each of the OCT volumes in this dataset for the aforementioned four AMD high-risk biomarkers, that is, DV, IHRF, SDD, and hDC. We also annotated these volumes using the same SLIViT model we trained on the 691 Houston dataset volumes. Figure 5 and S3 summarize respectively the true positive rate (TPR; also known as recall) vs. false positive rate (FPR; also known as false alarm rate) and the positive predictive value (PPV; also known as precision) vs. recall of SLIViT and the seven junior clinicians over the Pasadena Dataset. Clinicians typically reached comparable performance but had to invest 5,000-fold more time to do so (on average, it took 17 working hours net for each clinician to procure the annotations while SLIViT completed the job in under 12 seconds). Interestingly, SLIViT obtained considerably lower performance in the hDC classification task compared to the other biomarker classification tasks. A possible reason is the absence of a universal consensus on the clinical definition of hDC. This feature had the highest senior-specialists’ annotation discordance among the four biomarkers, suggesting indeed that it is harder to distinguish between cases and normals.

### SLIViT is robust to within-volume frame permutation

We next sought to explore SLIViT’s robustness to changes in the order of the frames encoding a volume. To this end, we generated 100 copies of the Houston Dataset and randomly shuffled each volume (in each of these 100 copies). Then, we used the same split to train 100 SLIViT models (one per shuffled copy; henceforth “shuffled models”) and one model on the Houston Dataset using the original order (henceforth “original model”) to classify the aforementioned structural AMD high-risk factors. Figure S4 shows the average bootstrapped ROC AUC dispersion of these 101 models. Interestingly, the original model did not outperform the shuffled models. We observed that compared to the 100 shuffled-models performance, the average rank of the original model across the four AMD biomarkers was 40. This finding suggested that even if the original order is not documented, SLIViT’s performance does not deteriorate. Thus, not only does SLIViT effectively aggregate information across slices, it can do this even when the order of slices is not maintained.

### The utility of 2D B-scan OCT in pre-training

The utility of ImageNet pre-training (henceforth “ImageNet weights”) has been demonstrated in various medical-imaging learning tasks^7,12,14,15,60–62^. That said, transfer learning between unrelated domains remains fairly controversial^18,63–65^. Moreover, commonalities across data modalities may be counterintuitive. We thus conducted a pre-training ablation study across the different learning tasks to evaluate the benefit of our cross-modality and cross-dimensionality transfer learning and assess the contribution of different selections made for the pre-training step of SLIViT (Figures S5 and S6). We compared four different initializations: random weights, ImageNet weights, random weights initialization followed by 2D OCT B-scans pre-training (henceforth “Kermany weights”), and ImageNet weights initialization followed by 2D OCT B-scans pre-training (henceforth “combined weights”). Of note, combined weights is the original initialization approach we intended (and eventually selected) for SLIViT. The results of this experiment indicate three key insights. First, we observed that using ImageNet weights improved performance for all the data modalities we tested relative to random weights. We also see that utilizing 2D OCT B-scans in pre-training (either Kermany weights relative to random weights or combined weights relative to ImageNet weights) improved performance in all downstream learning tasks. Interestingly, in the four OCT-related classification tasks, using Kermany weights (that is, without ImageNet) was the best approach and typically led to better performance, even when compared to the combined approach (Fig. S5). This last finding aligns with a conclusion previously indicated by Zhang Y., et al.^18^ and may suggest an even broader conclusion: for an out-of-distribution medical imaging task, pre-training using both (out-of-distribution) natural images and out-of-distribution medical images leads to better representation, when compared to pre-training only on out-of-distribution medical images (Fig. S6). On the other hand, for an in-distribution downstream task, pre-training only on in-distribution medical images is more beneficial (Fig. S5).

We also wished to assess the benefit of using supervised learning for pre-training, as opposed to self-supervised learning. The latter was demonstrated as a powerful approach in different visual tasks^66^, specifically, in the medical-imaging domain where procuring annotations is laborious and expensive^7,17,19,20^. We thus sought to explore the utility of self-supervised-based pre-training approach on SLIViT using an unlabeled version of the 2D OCT B-scans dataset (Figures S5 and S6). To this end, we took the REMEDIS approach^7^ that was originally shown to obtain remarkable performance when pre-trained even on much smaller (unlabeled) datasets than our 2D OCT B-scans dataset. Yet, initializing SLIViT with the fully supervised pre-trained weights significantly outperformed the self-supervised initialization in all downstream learning tasks (paired t-test p-value<0.001).

Interestingly, in both ultrasound and MRI experiments, SLIViT achieved superior performance relative to all competitor benchmarks tested, regardless of the pre-training strategy (Figures 2 and S6). This discovery further demonstrates the advantage of SLIViT’s architecture for out-of-distribution volumetric-medical-imaging learning tasks. For the in-distribution medical imaging task, that is, the (3D) OCT experiment, only pre-training strategies that leveraged the 2D OCT B-scan dataset at full, i.e., Kermany weights and combined weights, showed consistent superior performance relative to all other tested benchmark methods (left panels of Figures 3 and S1, and Fig. S5). This outcome was less surprising and corresponded with a previous study’s^18^ conclusion regarding the utility of in-distribution pre-training.

## Discussion

Procuring tens of thousands of annotated 3D biomedical-imaging samples to train standard 3D vision models is expert-time prohibitive, impeding the full optimization of such models. In this work we devised SLIViT, an AI-based framework that allows an accurate analysis of potentially any 3D biomedical-imaging dataset. SLIViT leverages a unique combination of deep vision modules and ‘prior knowledge’ from the 2D domain. This, in turn, allows it to be adept at 3D-biomedical-imaging-learning tasks, in which the number of annotated training samples is typically very limited, and significantly outperform domain-specific state-of-the-art models.

To showcase SLIViT’s effectiveness and generalizability we evaluated it over several classification and regression problems in diverse biomedical domains (retinal, cardiac, and hepatic) across different 3D biomedical-imaging data modalities (OCT, echocardiograms, and MRI) against domain-specific^24,27–29^ and generic (fully-supervised-^24,25^ and self-supervised-based^7,19^) state-of-the-art methods. We started by demonstrating SLIViT’s superiority when trained on less than 700 volumes in four independent binary classification learning tasks of retinal-disease risk factors with two independent 3D OCT datasets. Then we showed SLIViT’s superiority in two heart function analysis tasks both done with an echocardiogram dataset. We next tested SLIViT on an MRI dataset of 3D liver scans labeled with a corresponding hepatic fat content measurement and again, observed significant improvement compared to the state-of-the-art. We also showed that SLIViT was able to obtain on-par performance to clinical specialists’ assessment, but rather, almost four orders of magnitude faster compared to the annotation procurement net time required by the specialists. Lastly, we explored SLIViT’s learning ability robustness to randomly permuted volumes. We showed that a scenario of shuffled volumes dataset, a recurring situation in the very limited number of publicly available volumetric datasets, has little to no effect on SLIViT’s performance, meaning that SLIViT is potentially agnostic to imaging protocol.

To facilitate reproducibility, generalizability, and the likelihood that other researchers will be able to successfully apply SLIViT to their datasets, we intentionally avoided complex hyperparameter tuning and the usage of specialized hardware for training as required by other methods (e.g., ^19^). The sizes of the different architectures we used were set according to our available (standard) computational resources, and other hyperparameters were set to default values. This suggests that there is room for further improvement in task-specific performance. Yet, in its current form, SLIViT can serve as a reliable baseline model for any study of volumetric biomedical imaging. We believe that SLIViT’s simplicity is one of its major strengths.

The utility of self-supervised pre-training has been validated in numerous medical imaging learning tasks^7,19,20,67,68^, however, its general translatability across domains remains unclear^22^. According to our study, where a large-enough 2D labeled dataset is accessible and limited labeled volumes are available, the supervised pre-training approach is superior. This finding was supported by our experiments for fine-tuning both in the same domain and across domains. That being said, as demonstrated, SLIViT’s pre-training strategy is very flexible and can thus harness the utility of self-supervised approaches, such as REMEDIS. If one has access to an(other) unlabeled dataset of relevant medical images (whether 2D or 3D), then self-supervised pre-training SLIViT (either) as an alternative to (or followed/preceded by) supervised 2D OCT B-scans pre-training may further improve the model’s performance. Notably, the end-to-end fine-tuning approach SLIViT takes (see Methods) was shown to attain typically better performance for self-supervised-based medical-imaging learning tasks^22^. That is, SLIViT already employs an optimized fine-tuning approach for a potential self-supervised-based avenue.

SLIViT was tested on 3D OCT scans, echocardiograms, and MRI volumes and can potentially be leveraged to analyze other types of data modalities, such as 3D CT scans and 3D X-ray imaging. Such biomedical volumetric imaging data is inherently structured in the sense that they involve a limited assortment of objects and movements (typically shrinkage, dilation, and shivering). SLIViT is specifically tailored to be adept at analyzing a series of biomedical frames created in a structured biomedical-imaging process and does not pretend to be proficient at learning problems of natural videos, such as action recognition tasks. Natural videos are inherently more complex, as the background may change, objects may flip, change color (due to shade), and even disappear (due to obfuscation), let alone when considering a multi-scene video. In addition, there is a plethora of gigantic natural video datasets that allow standard 3D-based vision models to be adequately tuned for natural video learning tasks. We thus do not expect SLIViT to outperform (as is) standard 3D-based vision models in natural-videos-learning tasks (such as action recognition). That being said, SLIViT could potentially be tweaked to perform well on natural videos as well, e.g., using a different feature-map extractor, however, this direction requires further research.

Importantly, there are multiple additional steps that are required in order to deploy SLIViT in a clinical setting. Notably, the point of operation (tradeoff between precision and recall) is application specific and further optimization may be required to obtain optimal results at that point of operation. We note that point of operation varies also across clinicians (see Figures 5 and S3). Moreover, additional evaluations of the models are required to ensure no systematic biases exist that would lead to increasing health disparities^69^.

Overall, this study highlights an important step toward fully automating volumetric-biomedical-imaging annotation. The major leap happens under ‘real life’ settings of a low-number training dataset. SLIViT thrives given just hundreds of training samples for some tasks giving it an extreme advantage over other 3D-based methods, in almost every practical case that is related to 3D biomedical-imaging annotation. Even under the unrealistic assumption that the financial resources are endless, in ongoing research, due to its nature, the hurdle of a limited-size training dataset is inevitable. Once a previously unknown disease-related risk factor is found and characterized, it could take months in order to train a specialist to be able to accurately annotate this recently-discovered risk factor in biomedical images at scale. However, using a relatively small training dataset (that can be annotated within only a few working days of a single trained clinician), SLIViT could dramatically expedite the annotation process of many other non-annotated volumes with an on-par performance level of a clinical specialist.

## Methods

### SLIViT’s development and analysis

SLIViT was implemented in Python 3.8 using PyTorch^70^ v1.10.2, fast.ai^71^ v2.6.3, and scikit-learn^72^ v1.0.2 libraries (full libraries and version list can be found at https://github.com/berkindurmus/SLIViT/blob/main/requirements.txt).

### Model specifications

The SLIViT framework contains a preprocessing step, a 2D ConvNeXt that serves as a feature-map extractor, and a vision transformer (ViT) that serves as a feature-map integrator (see Fig. 1). A ConvNeXt architecture has several complexities^39^. Here we used the backbone of the tiny variant (ConvNeXt-T) with 256×256 image size as SLIViT’s feature-map extractor. The ViT-based feature-map integrator underwent few adjustments with respect to the original architecture^40^, including using GeLu as the activation functions^73^ and initializing the positional embeddings as the number of the original slice. Notably, we intentionally avoid complex hyperparameter tuning and usage of specialized hardware as required by other methods^19^. The ConvNeXt’s variant (T) and the ViT’s depth (# layers = 5) were set according to our available (standard) computational resources to facilitate reproducibility, generalizability, and the likelihood that other researchers will be able to successfully apply it to their datasets. The ViT’s width is governed by the number of 2D frames of the input volume.

Let N be the number of H×W 2D frames of an input image. Given an input W×H×N image, its N frames are resized (according to the ConvNeXt-T variant) and tiled into an image of size N*256×256 (see Step (1) in Fig. 1). The manipulated image is then fed into the feature-map extractor which generates, in turn, an N*8×8 feature maps with F=768 filters each. These feature maps are then reshaped into N different 8×8×768 feature maps (see Step (3) in Fig. 1), each corresponding to a slice in the original volume. Each of the feature maps is flattened into an 8∗8∗768 (1D) vector and tokenized into a vector of size 768 using a fully connected (FC) layer. The bias term of the FC layer is initialized as the feature-map number (that essentially corresponds to an original slice number), and the projected feature volumes are then fed into the ViT (along with a class token of the same size). The ViT outputs N encoded values and a class token. The class token is then fed into another FC layer to generate final output. Using the 2D ViT as a feature-map integrator corresponds with the Factorised Encoder with ‘late fusion of depth information’ of the previously devised 3D ViT named ViViT^25^, yet, is far less complex than the 3D ViT.

### Pre-training

We borrowed an ImageNet-1K pre-trained SLIViT-like feature-map extractor architecture, i.e., a ConvNeXt-T backbone, from https://huggingface.co/facebook/convnext-tiny-224, and appended to it a subsequent FC layer to fit a four-category classification task. We then trained this SLIViT-backbone-like module on the publicly available labeled Kermany Dataset^41,74^. Training the feature-map extractor on the Kermany Dataset took less than 12 hours using a single NVIDIA Tesla V100 Volta GPU Accelerator 32GB Graphics Card. Several sets of pre-trained weights were examined in this study (see The utility of 2D B-scan OCT in pre-training section). The pre-trained backbone weights obtained from combining ImageNet initialization with additional pre-training on the Kermany Dataset (henceforth “combined weights”), which typically led to the best performance, are available at project’s GitHub repository (see Code availability section).

### Per-task fine-tuning

Each of the SLIViT models used in the different experiments reported here, was initialized with the combined weights. The fine-tuning was done in an end-to-end fashion^22^. Namely, rather than merely training the downstream feature-map integrator, while keeping the feature-map extractor frozen, all the model’s parameters were set as trainable, and were then fine-tuned (according to the dataset and task in question). Notably, we intentionally avoided complex hyperparameter tuning as required by some other methods (e.g., ^19^) to facilitate reproducibility and generalizability. Frames were resized into 256×256 pixels to fit SLIViT’s backbone architecture and then, standard preprocessing transformations were applied (including contrast stretching, random horizontal flipping, and random resize cropping) using PyTorch’s default values. Binary cross entropy and L1 norm were used as loss functions for the classification and regression tasks, respectively. In each experiment, excluding the ultrasound (in which the split was given), a random validation set was used for determining the convergence of the training process with the same loss function metric used for the test set evaluation. The model was optimized using the default fast.ai optimizer with the default parameters. The starting learning rate in each training procedure was chosen by fast.ai’s learning rate finder and the model was fitted using the fit-one-cycle approach for faster convergence^75,76^. All models were trained with four samples per batch and early stopping was set to five epochs, meaning that the training process continued until no improvement was observed in the validation loss for five consecutive passes on the whole training set. The model weights that achieved the lowest loss on the validation set during training were used for the test set evaluation. Weights & Biases^77^ was used for experiment tracking and visualizations of the training procedures.

### Statistical analysis

The performance of each trained model was evaluated (on the corresponding test set) using an appropriate metric score. The binary classification tasks were evaluated using area under the ROC and PR curves. The regression tasks were evaluated using the *R*^2^ metric. The test set predictions were calculated and a 90% confidence interval (CI) was computed for each evaluated score using a standard bootstrapping procedure with 1,000 iterations as done in other studies^17,78^. Briefly, let *n* denote the test set size, for each bootstrap iteration *n* samples were randomly drawn (with repetition) and based on the predictions of the sampled set a single score was obtained. Out of the 1,000 sampled-sets score distribution, the 50th and 950th ranked scores were selected to obtain the 90% CI. In order to compute the significance value of the difference between two given distributions (induced by two different models) a paired t-test on the distribution of differences between the sampled-set corresponding scores was computed $\left(H_{A}:\mu \neq 0\right).$ SLIViT’s performance improvement was considered to be significant if the paired t-test produced a p-value lower than 1e-3 subject to Bonferroni correction for multiple hypothesis testing.

### Datasets

#### The Houston Dataset

1,128 patients were diagnosed with intermediate AMD in their scanned eye by clinical examination (Beckman Classification^79^) at the Retina Consultants of Texas Eye Clinics between October 2016 and October 2020. This study was reviewed and approved by the Ethics Committee of Retina Consultants Texas (Houston Methodist Hospital, Pro00020661:1 “Retrospective Prospective Analysis of Retinal Diseases”). As the data collection was retrospective, a waiver of informed consent was granted. In case both eyes of a given patient were eligible, one eye was randomly included in the dataset. The dataset included Heidelberg Spectralis (HRA+Optical Coherence Tomography OCT SPECTRALIS; Heidelberg Engineering, Inc, Heidelberg, Germany) 6×6 mm (fovea centered, 10X10 degrees; 49 B-scans spaced 122 microns apart, ART=6) OCT volumes. The data were transferred to the Doheny Image Reading Research Laboratory (DIRRL) for imaging analysis and annotation of the structural OCT biomarkers for AMD progression^80,81^. The AMD-biomarker analysis was conducted at the Doheny Image Reading Research Laboratory (DIRRL) in compliance with the Declaration of Helsinki and approved by the UCLA Institutional Review Board (IRB, Ocular Imaging Study, Doheny Eye Center UCLA). Cases with evidence of late stage of AMD and/or additional macular diseases or poor-quality imaging were excluded from the analysis. In total, 691 eyes (of 691 patients) were eligible for the biomarkers analysis. The annotations were procured by a senior clinical retina specialist. The recorded case frequency in the whole dataset was as follows: (1) 48.23% of the scans had drusen volume > 0.03 *mm*^*3*^ within the 3 central mm^2^ (denoted DV); (2) 36.17% of the scans had intraretinal hyperreflective foci (denoted IHRF); (3) 31.45% of the scans had subretinal drusenoid deposits (SDD); and (4) 11.27% of the scans had hyporeflective drusen core (hDC). Of note, some scans were positive for more than one biomarker.

#### The SLIVER-net Dataset

The SLIVER-net Dataset, which was originally used by Rakocz and others^27^ to tune and validate SLIVER-net, was collected from three independent medical centers between February 2013 and July 2016^82^. The dataset consisted of 1,007 OCT volumes each consisting of 97 B-scans (97,679 B-scans overall) collected from 649 subjects of the Amish general population, who had a record of at least one individual with AMD in the family history. Imaging was conducted at three clinical centers in Pennsylvania, Indiana, and Ohio under the supervision of investigators at the University of Pennsylvania (UPEN), University of Miami (MU), and Case Western Reserve University (CWRU), respectively. The research was approved by the institutional review boards (IRBs) of the respective institutions and all subjects signed written informed consent. All OCT B-scan volumes in this dataset were acquired with the Heidelberg Spectralis OCT using a scan pattern centered on the fovea (20°×20°; 97 B-scans; 512 A-scans per B-scans; ART 9). In order to fit the Houston Dataset trained model, we down-sampled each of the SLIVER-net Dataset volumes by taking every other B-scan, thus squeezing each volume to 49 B-scans. Also, to avoid aliasing, we applied an anti-aliasing filter on OCT volumes.

The positive-label frequencies in this dataset were 3.37%, 7.87%, 2.0%, and 2.67%, for DV, IHRF, SDD, and hDC, respectively. Although the annotations for this dataset included the eyes laterality, the scans themselves lacked the laterality obscuring the link between a scan to its annotation in case both eyes were scanned for a patient. To address this gap, we considered the middle slice per volume to determine the laterality and trained a standard CNN on the Houston Dataset (that had the eyes laterality recorded). Using the trained network (97% accuracy on an external test set; not shown) we inferred the laterality for the SLIVER-net dataset scans when needed, that is, when both eyes of the same patient were scanned.

#### The Pasadena Dataset

The Pasadena Dataset established for this study contained 205 3D OCT B-scan volumes (fovea centered, 10×10 degree, ART=5) collected from 205 individuals at the Doheny-UCLA Eye Centers in Pasadena between 2013 and 2022. This study was reviewed and approved by the IRB of the University of California, Los Angeles (UCLA IRB # 15–000083). Informed consent was waived for study participants given the retrospective nature of the study. Each of the OCT volumes was acquired on the Heidelberg Spectralis HRA+Optical Coherence Tomography (OCT SPECTRALIS; Heidelberg Engineering, Inc, Heidelberg, Germany). Out of the 205 OCT volumes, 198 contained 97 B-scans and seven contained 49 B-scans. The OCT B-scans were independently annotated by ten DIRRL-certified clinical retina specialists: three seniors (expert retina specialists) and seven juniors. The ground truth for this dataset was determined by the senior retina specialists. Although the senior graders agreed in most cases, in the atypical case of disagreement, the ground truth was obtained by a majority vote of the senior graders’ quorum. The positive-label frequencies in this dataset were 32.8%, 51.6%, 42.9%, and 12.5%, for DV, IHRF, SDD, and hDC, respectively.

#### The EchoNet-Dynamic Dataset

The EchoNet-Dynamic Dataset^48^ was downloaded on September 7, 2022. The dataset contains 10,030 echocardiograms (heartbeat ultrasound videos) obtained from 10,030 different individuals who underwent echocardiography between 2006 and 2018. Each echocardiogram was labeled with a continuous number (between zero and one) representing the ejection fraction (EF). The EF was obtained by a registered sonographer and further verified by a level 3 echocardiographer. The minimal EF in the dataset was 0.069 while the maximal was 0.97. The average EF was 0.558 with a standard deviation of 0.124. The dataset already set a random split for train, validation, and test sets of sizes 7,465 (74.43%), 1,288 (12.84%), and 1,277 (12.73%), respectively. In contrast to the other datasets used in this study, the number of frames (2D images) per video in the dataset was not constant but rather varied from 28 to 1,002 (with nearly 177 frames on average and a standard deviation of 58 frames). To standardize the data we followed the same approach that the EchoNet paper authors took and sampled 32 equally-spaced frames per volume.

#### The United Kingdom Biobank Dataset

The United Kingdom Biobank (UKBB) Dataset of MRI imaging with Proton Density Fat Fraction (PDFF) measurements was downloaded on June 7, 2022, from the UKBB repository^23^. The UKBB is a widely studied population-scale repository of phenotypic and genetic information for roughly half a million individuals. At the time of the study, the UKBB made available 16,876 PDFF measurements acquired from a subset of the 54,606 total hepatic-imaging MRIs. The MRI data of each individual consisted of an unordered series of 36 imaging scans in DICOM format at 284 by 288 resolution (in-plane pixel spacing 9.3 mm) acquired from a single breath-hold session. Of the data available, we identified a subset of 9,954 White British individuals who were unrelated and possessed both the hepatic MRI and PDFF measurement. The individuals were further divided into train, validation, and test sets of sizes 5972 (60%), 1991 (20%), and 1991 (20%), respectively.

## Supplementary Material

Supplement 1

## Data Availability

The Kermany dataset was downloaded from https://www.kaggle.com/datasets/paultimothymooney/kermany2018. The 3D OCT B-scan data are not publicly available due to institutional data use policy and concerns about patient privacy. However, they are available from the authors upon reasonable request and with permission of the institutional review board. The echocardiogram dataset was downloaded from https://echonet.github.io/dynamic/index.html#dataset. The MRI dataset was downloaded from https://www.ukbiobank.ac.uk under application number 33127.

## Footnotes

Code availability The code of SLIViT is available at the project’s GitHub repository: https://github.com/berkindurmus/SLIViT.
