# Supplementary material for: SLIViT: a general AI framework for clinical-feature diagnosis from limited 3D biomedical-imaging data
Source: Res Sq. 2023 Nov 21:rs.3.rs-3044914. Preprint. [Version 2] doi: 10.21203/rs.3.rs-3044914/v2 (PMC10690310; doi:10.21203/rs.3.rs-3044914/v2)
Supplement: Supplement 1 [file NIHPPrs3044914v2-supplement-1.pdf]

Supplementary Material

Figure S1 | PR-AUC performance comparison of five models in four independent AMD-biomarker classification tasks when trained on less than 700 OCT volumes

Shown are the PR AUC scores as an alternative scoring metric for the experiment shown in Figure 3. The dashed lines represent the corresponding biomarker's positive-label prevalence, which is the expected PR AUC score of a naive classifier. The left panel shows the performance when trained and tested on the Houston Dataset. The right panel shows the performance when trained on the Houston Dataset and tested on the SLIVER-net Dataset (see Table S1B). Box plot whiskers represent a 90% CI.

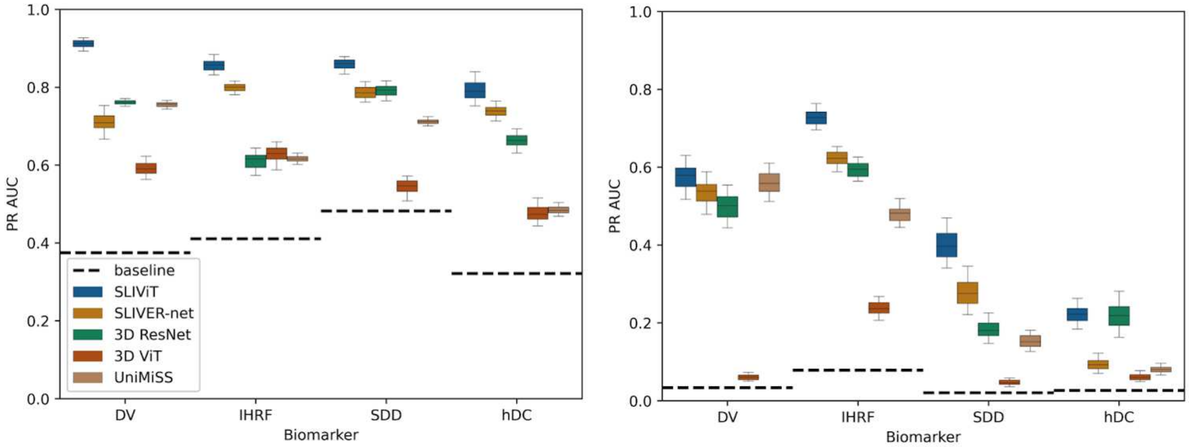

Figure S2 | Performance comparison of a cardiomyopathy binary classification task on echocardiograms

Shown are the PR curves yielded by modeling SLIViT (blue) and 3D ResNet (green) to classify cardiomyopathy. The shaded areas represent a 90% CI.

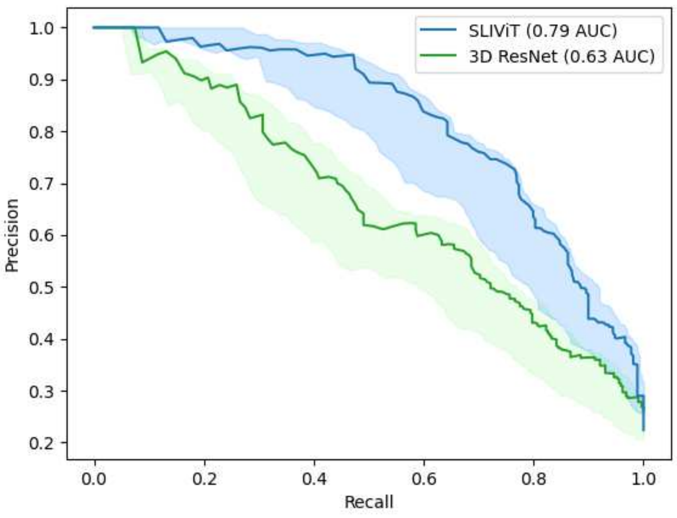

Figure S3 | SLIViT's PR performance compared to junior clinical retina specialists' assessment

Shown are the PR curves (blue) of SLIViT trained to predict four AMD high-risk biomarkers (DV, IHRF, SDD, and hDC; see main text) using less than 700 OCT volumes (Houston Dataset) and tested on an independent dataset (Pasadena Dataset). The light-blue shaded area represents a 90% CI for SLIViT's performance. The red dot represents the specialists' average performance. The green asterisks correspond to the retina specialists' assessments. Two of the clinical specialists obtained the exact same performance score for IHRF classification.

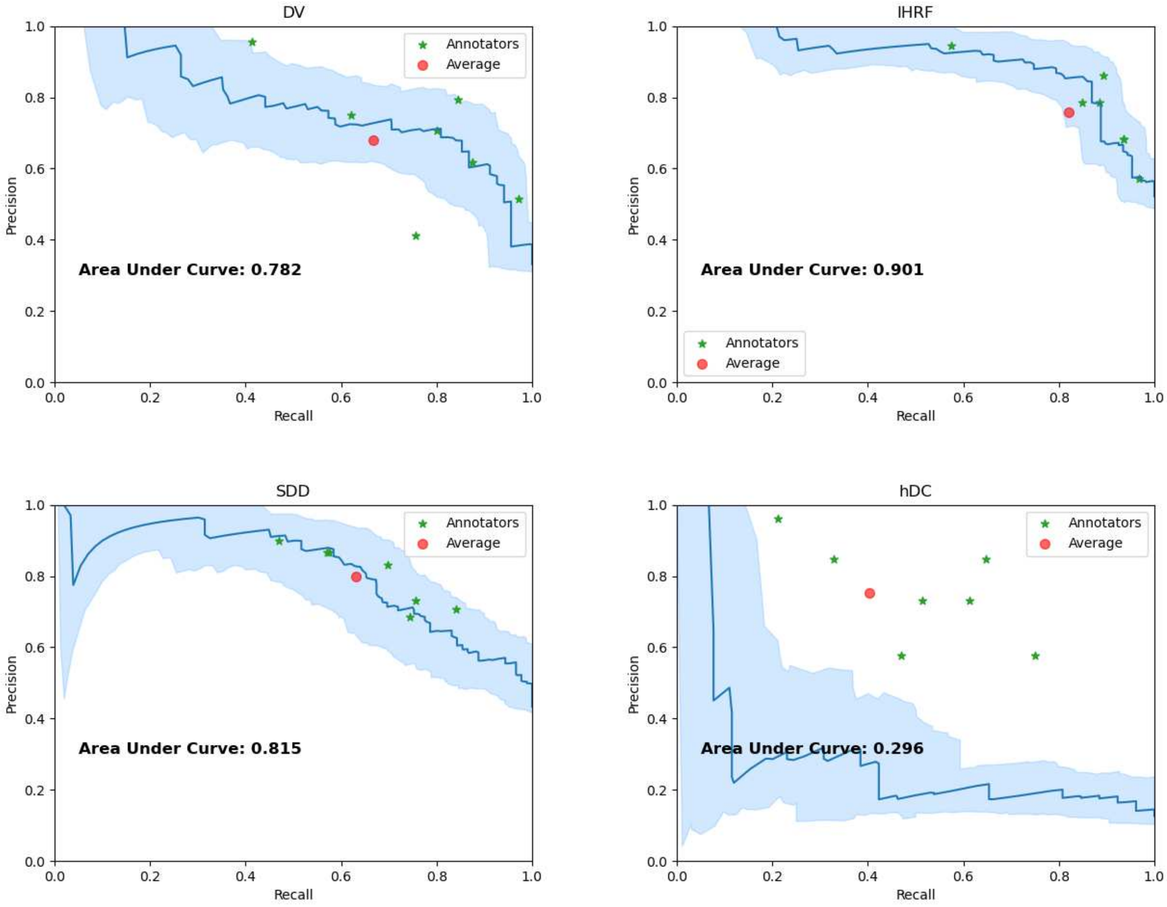

Figure S4 | SLIViT's performance in a volumetric-OCT frame-permutation experiment

Shown is the ROC AUC scores distribution of 100 shuffled models (light blue) trained on 100 different (shuffled) copies of a volumetric-OCT dataset. The expected performance of a naive classifier is 0.5. Box plot whiskers extend to the 5th and the 95th percentiles of the 100 shuffled models' performance distribution. The dashed blue line represents the performance of a SLIViT model trained on the volumetric-OCT dataset using the original order of each volume. The performance ranks of this latter model compared to the former models' distribution were 22, 34, 56, and 47 for DV, IHRF, SDD, and hDC, respectively.

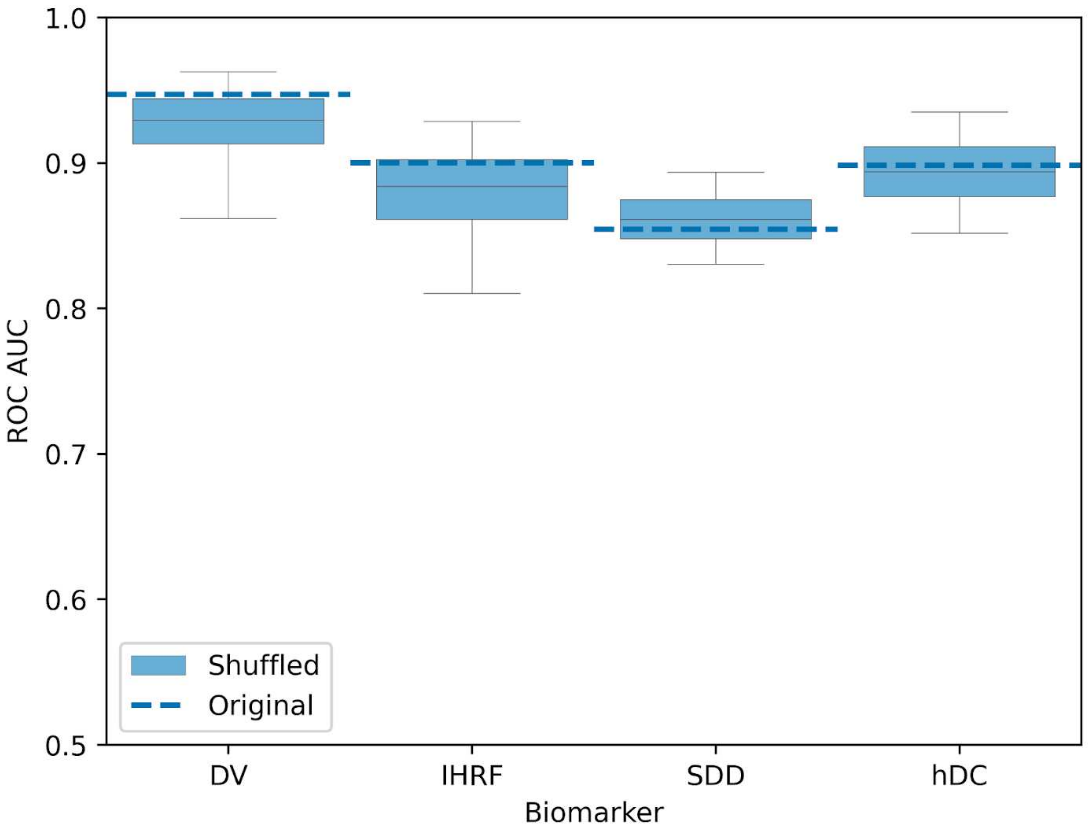

Figure S5 | Pre-training ablation study for (volumetric) OCT-related downstream learning tasks

Shown are the ROC (left) and PR (right) AUC scores across different fine-tuned models for volumetric-OCT classification tasks initialized with five different sets of pre-trained weights. The expected ROC AUC score of a naive classifier is 0.5. Combined, the proposed SLIViT's initialization, is ImageNet weights initialization followed by supervised pre-training on the Kermany Dataset. ssCombined is an ImageNet weights initialization followed by self-supervised pre-training on an unlabeled version of the Kermany Dataset. The dashed lines represent the corresponding biomarker's positive-label prevalence, which is the expected PR AUC score of a naive classifier. Box plot whiskers represent a 90% CI.

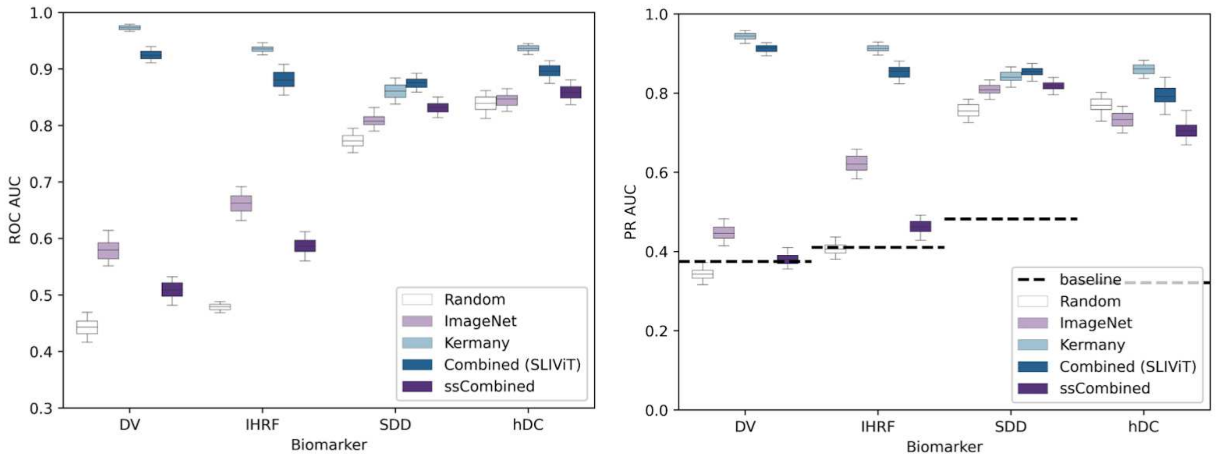

Figure S6 | Pre-training ablation study for (volumetric) non-OCT-related downstream learning tasks

Shown are the R^2 scores for the volumetric ultrasound and MRI regression tasks initialized with five different sets of pre-trained weights. Combined, the proposed SLIViT's initialization, is ImageNet weights initialization followed by supervised pre-training on the Kermany Dataset. ssCombined is an ImageNet weights initialization followed by self-supervised pre-training on an unlabeled version of the Kermany dataset. Box plot whiskers represent a 90% CI.

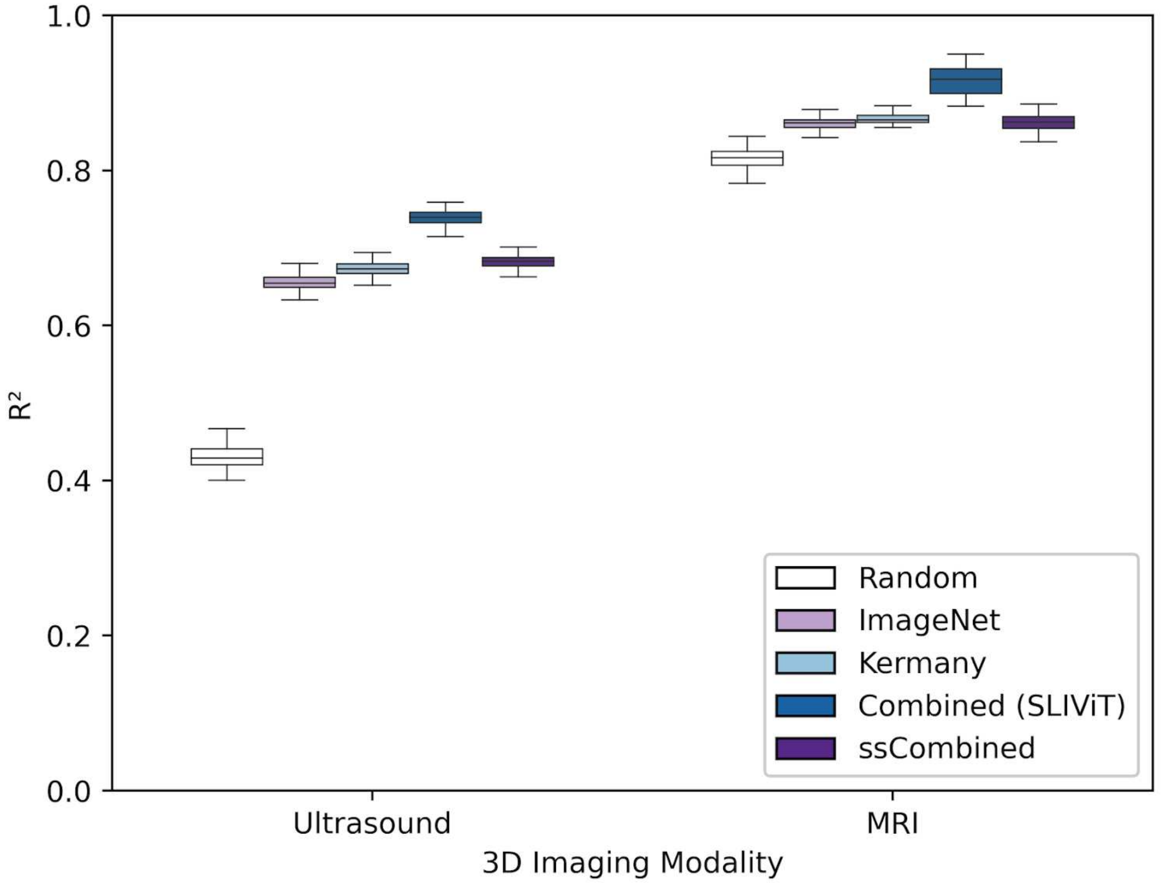

Table S1 | Average classification performance scores of SLIViT, SLIVER-net, 3D ResNet, 3D ViT, and UniMiSS trained on less than 700 OCT volumes

Shown are the performance raw numbers underlying Fig. 3 (ROC AUC) and Fig. S1 (PR AUC) of the AMD high-risk biomarker prediction experiments. The numbers in the square brackets represent the corresponding 90% CI.

A – ROC AUC scores

Test dataset	Method	DV	IHRF	SDD	hDC
Houston	SLIViT	.924 [.909, .938]	.883 [.86, .906]	.877 [.855, .893]	.89 [.877, .916]
	SLIVER-net	.838 [.813, .86]	.837 [.82, .855]	.805 [.78, .827]	.854 [.836, .869]
	3D ResNet	.777 [.769, .783]	.655 [.625, .682]	.783 [.762, .806]	.782 [.757, .805]
	3D ViT	.576 [.547, .605]	.617 [.583, .651]	.629 [.598, .66]	.667 [.63, .703]
	UniMiSS	.783 [.771, .793]	.675 [.66, .69]	.714 [.701, .726]	.715 [.7, .729]
SLIVER-net	SLIViT	.958 [.941, .975]	.891 [.873, .909]	.967 [.959, .973]	.863 [.839, .892]
	SLIVER-net	.933 [.919, .95]	.839 [.817, .86]	.911 [.9, .922]	.625 [.576, .676]
	3D ResNet	.904 [.891, .911]	.8 [.788, .813]	.895 [.865, .925]	.716 [.689, .737]
	3D ViT	.642 [.611, .674]	.758 [.737, .78]	.735 [.7, .77]	.718 [.677, .758]
	UniMiSS	.929 [.915, .939]	.781 [.753, .808]	.774 [.723, .825]	.795 [.765, .825]

B – PR AUC scores

Test dataset	Method	DV	IHRF	SDD	hDC
Houston	SLIViT	.914<br>[.898, .928]	.852<br>[.826, .875]	.855<br>[.831, .879]	.795<br>[.747, .838]
	SLIVER-net	.708<br>[.676, .744]	.799<br>[.778, .817]	.785<br>[.752, .816]	.74<br>[.716, .76]
	3D ResNet	.759<br>[.748, .769]	.619<br>[.584, .647]	.791<br>[.77, .815]	.669<br>[.622, .697]
	3D ViT	.589<br>[.551, .628]	.627<br>[.584, .67]	.54<br>[.494, .586]	.479<br>[.428, .529]
	UniMiSS	.755<br>[.742, .769]	.616<br>[.598, .634]	.711<br>[.696, .726]	.484<br>[.462, .506]
SLIVER-net	SLIViT	.575<br>[.517, .63]	.728<br>[.696, .763]	.399<br>[.341, .469]	.222<br>[.184, .263]
	SLIVER-net	.535<br>[.47, .588]	.621<br>[.588, .653]	.278<br>[.221, .345]	.093<br>[.07, .122]
	3D ResNet	.497<br>[.444, .553]	.593<br>[.563, .626]	.183<br>[.147, .225]	.219<br>[.162, .282]
	3D ViT	.06<br>[.046, .074]	.238<br>[.199, .276]	.046<br>[.032, .061]	.061<br>[.042, .08]
	UniMiSS	.56<br>[.497, .623]	.48<br>[.431, .528]	.153<br>[.114, .191]	.08<br>[.061, .099]
